# Supplementary material for: Ultrasensitive, high-dynamic-range and broadband strain sensing by time-of-flight detection with femtosecond-laser frequency combs
Source: Sci Rep. 2017 Oct 17;7:13305. doi: 10.1038/s41598-017-13738-w (PMC5645398; doi:10.1038/s41598-017-13738-w)
Supplement: Supplementary file 1 — Supplementary Information [file 41598_2017_13738_MOESM1_ESM.pdf]

Supplementary Information for "Ultrasensitive, high-dynamic-range and broadband strain sensing by time-of-flight detection with femtosecond-laser frequency combs"

Xing Lu, Shuangyou Zhang, Xing Chen, Dohyeon Kwon, Chan-Gi Jeon, Zhigang Zhang, Jungwon Kim, and Kebin Shi

1. Fibre-loop optical-microwave phase detector (FLOM-PD)

Figure S1 shows the schematic of the FLOM-PD [S1, S2]. It has a Sagnac-loop interferometer structure consisting of a polarization-maintaining (PM) 3-dB coupler, a PM circulator, a unidirectional high-speed $LiNbO_3$ phase modulator, a non-reciprocal quarter-wave bias unit, and a balanced photodetector. The non-reciprocal quarter-wave bias unit is used to bias the interferometer at the balanced point. When a microwave signal with an integer multiple of the repetition rate of MLL is applied to the unidirectional phase modulator in the FLOM-PD, the copropagating pulses experience the microwave phase modulation while the counterpropagating pulses do not. Therefore, there is an intensity difference between the two outputs of the loop, which is proportional to the temporal position displacement between the light pulse and the microwave zero crossing. More detailed information on the design and performance of FLOM-PDs can be found in [S1] and [S2].

By using FLOM-PDs, we can precisely synchronize the microwave signal with optical pulse trains. The typical out-of-loop residual phase noise power spectral density is shown in Figure S2. The locking bandwidth is larger than 100 kHz. The

residual phase noise at 1 Hz (10 kHz) offset frequency is about -135 dBc/Hz (-156 dBc/Hz), which results in 177 attoseconds integrated RMS timing jitter from 0.01 Hz to 100 kHz offset frequency.

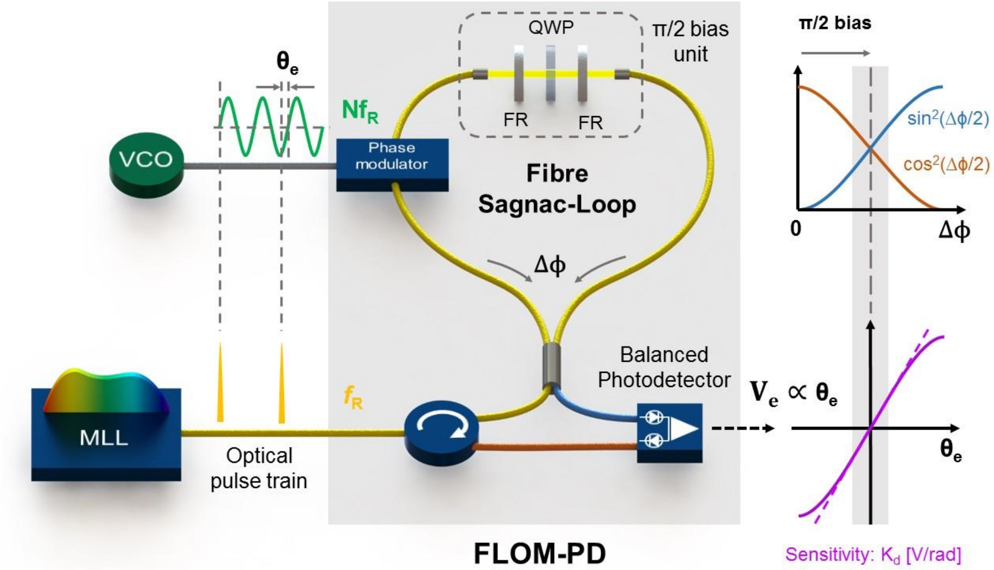

Figure S1. Schematic of the FLOM-PD. FR, Faraday rotator; QWP, quarter-wave plate.

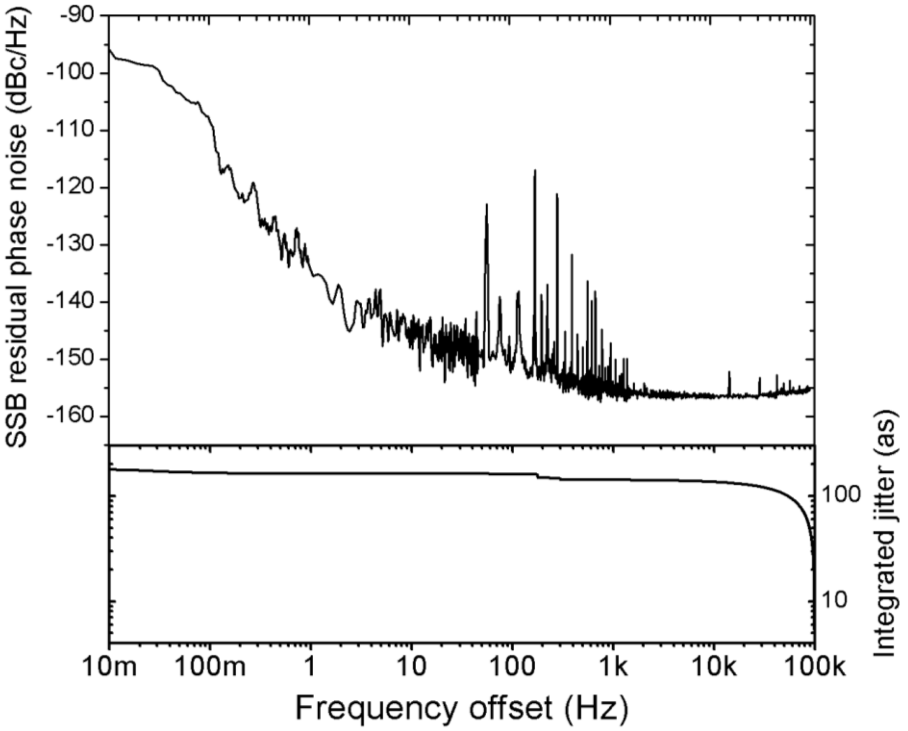

Figure S2. Out-of-loop residual phase noise from 0.01 Hz to 100 kHz. SSB, Single-sideband.

2. Fibre noise compensation for the remote strain sensing

In this strain sensing scheme, the strain information is encoded in the time-of-flight of optical pulse trains. Therefore, the timing drift induced by the connective fibre link degrades the strain resolution in the remote strain sensing. Figure S3 **a** and **b** show the experimental setup without and with compensation technique, respectively. Figure S4 shows the measured strain spectra (curves **a** and **b** correspond to without and with compensation, respectively), and it is clear that the WDM compensation method can improve the strain resolution by more than 100 times in the 0.1 Hz – 1 kHz frequency range.

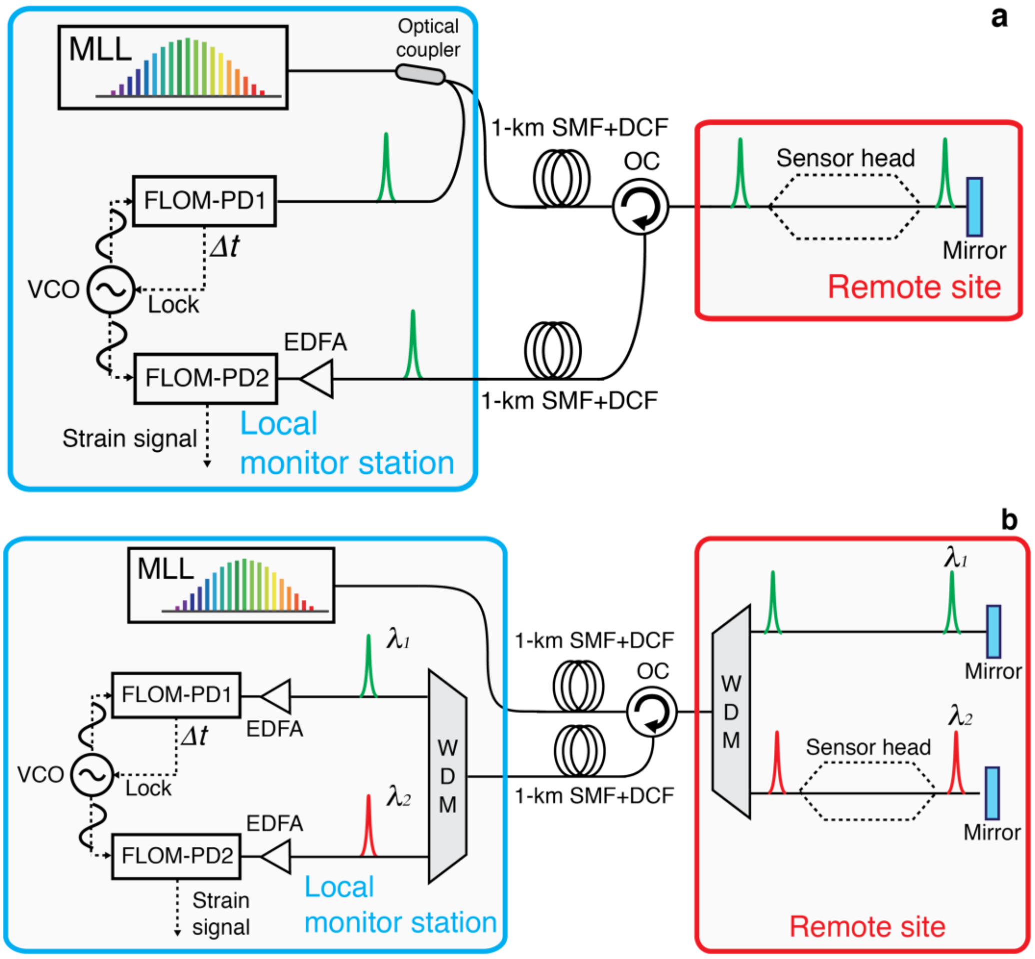

Figure S3. **a**, experimental setup without fibre noise compensation; **b**, experimental setup with WDM fiber noise compensation technique

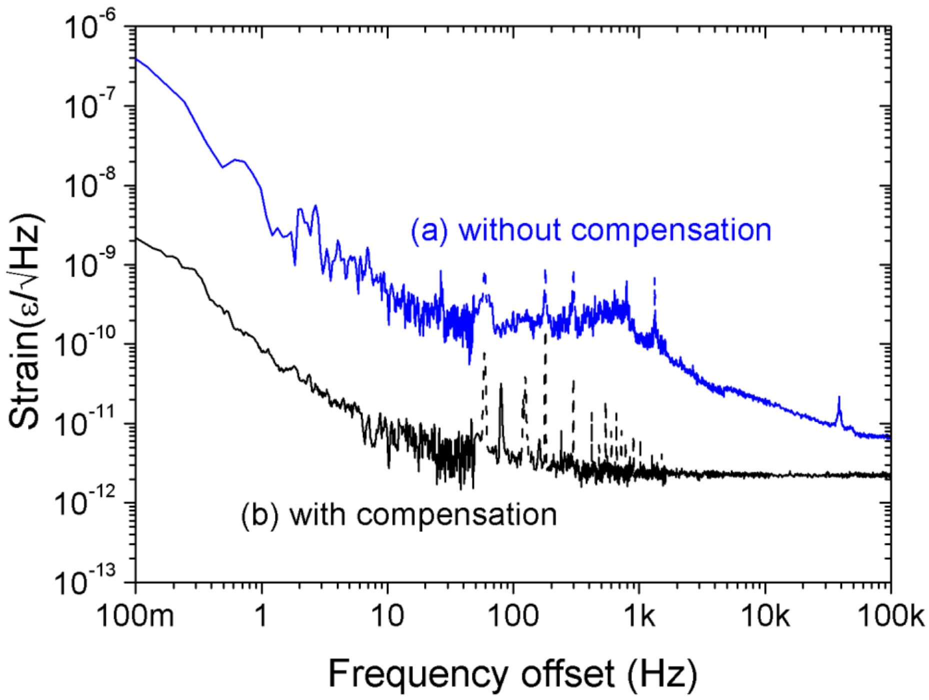

Figure S4. Strain power spectral density, curve (**a**) without fibre noise compensation, curve (**b**) with fibre noise compensation. Power supply peaks at 60 Hz and its harmonics are indicated as dashed lines.

References

S1. Jung, K. & Kim, J. Subfemtosecond synchronization of microwave oscillators with mode-locked Er-fiber lasers. *Opt. Lett.* **37**, 2958-2960 (2012).

S2. Kim, J., Jung, K., Shin, J., Jeon, C. & Kwon, D. Femtosecond Laser-Based Microwave Signal Generation and Distribution. *J. Lightwave Technol.* **34**, 4631-4638 (2016).
